# Spatial evolutionary public goods game on complete graph and dense complex networks

**DOI:** 10.1038/srep09381

**Published:** 2015-03-23

**Authors:** Jinho Kim, Huiseung Chae, Soon-Hyung Yook, Yup Kim

**Affiliations:** 1 Department of Physics and Research Institute for Basic Sciences, Kyung Hee University, Seoul 130-701, Korea

## Abstract

We study the spatial evolutionary public goods game (SEPGG) with voluntary or optional participation on a complete graph (CG) and on dense networks. Based on analyses of the SEPGG rate equation on finite CG, we find that SEPGG has two stable states depending on the value of multiplication factor *r*, illustrating how the “tragedy of the commons” and “an anomalous state without any active participants” occurs in real-life situations. When *r* is low (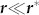), the state with only loners is stable, and the state with only defectors is stable when *r* is high (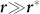). We also derive the exact scaling relation for r*. All of the results are confirmed by numerical simulation. Furthermore, we find that a cooperator-dominant state emerges when the number of participants or the mean degree, 〈*k*〉, decreases. We also investigate the scaling dependence of the emergence of cooperation on *r* and 〈*k*〉. These results show how “tragedy of the commons” disappears when cooperation between egoistic individuals without any additional socioeconomic punishment increases.

The emergence and evolution of cooperation is central to understanding the evolution and human activity-associated dynamics. One of the most popular theoretical frameworks that is used to shed light on such issues is evolutionary game theory. Game theory has also been successfully applied in diverse fields such as evolutionary biology and psychology^1^, computer science and operations research^2^,^3^, political science and military strategy^4^,^5^, cultural anthropology^6^, ethics and moral philosophy^7^, economics^8^,^9^, traffic flow research^10^,^11^ and public health^12^. When preferences and goals of participating agents are in conflict, game theory can explain and predict interactive decisions^13^. The central aim of game theory research is to determine conditions needed for cooperation to emerge between egoistic individuals^14^,^15^,^16^. Two of the most famous models for game theory include the prisoner's dilemma (PD) and public goods game (PGG)^17^. While the PD for a pairwise interaction attracted the attention of biologists and social scientists, PGG for group interactions was the focus of studies in experimental economics^18^. The PGG was often studied to identify effects of collective action arising from joint group decisions. Although sometimes the group interactions can be modeled as repeated simple pair interactions as with the PD, the most fundamental unit of the game is irreducible multi-agent nature^13^,^19^,^20^. The PGG offers valuable insight into prevailing socioeconomic problems such as pollution, deforestation, mining, fishing, climate control and environmental protection^13^. In identifying potential solutions to these issues, PGGs with various strategies^13^,^17^,^20^,^21^,^22^,^23^,^24^,^25^,^26^,^27^,^28^,^29^,^30^,^31^,^32^,^33^,^34^,^35^,^36^,^37^,^38^,^39^,^40^,^41^,^42^,^43^,^44^,^45^,^46^,^47^ have been suggested and studied. Economists have mainly studied PGG with two strategies, *C* and *D*, in which all agents participate and share a single common pool^21^,^22^,^23^,^24^.

In this report, we focus on a PGG with voluntary participation^25^ in which three strategic players (cooperators (*C*), defectors (*D*) and loners (*L*)) are considered. Each *C* contributes *c* to the common pool, whereas *D* attempts to exploit the resource at no cost. Then, each *C* gets the payoff *P_C_* as *P_C_* = *rcn_C_*/(*n_C_* + *n_D_*) − *c*, whereas each *D* obtains *P_D_* as *P_D_* = *rcn_C_*/(*n_C_* + *n_D_*). Here, *n_C_* (*n_D_*) denotes the number of *C*'s (*D*'s) participating in the game, and *r*(>1) is the multiplication factor, which describes synergistic effects of cooperation. In contrast, *L* refuses to participate in the game and relies only on private payoff *σ*. In this report the condition, 0 < *σ* < *c*(*r* − 1), is imposed^25^.

Recently, the spatial evolutionary PGG (SEPGG) has been intensively studied to understand how steady-state strategies emerge on various structures and to identify characteristic features of such steady-state strategies^17^,^25^,^26^,^28^,^29^,^30^,^31^,^32^,^33^. In the SEPGG, each agent is assigned to a node on a lattice or network. In a unit game of the SEPGG, only a randomly selected agent and its linked neighbors participate^26^. Then, in each update of the SEPGG, a randomly selected agent *i* adopts the strategy of a randomly selected neighbor *j* of *i* with a transition probability *f_ij_* that depends on payoffs *P_i_* and *P_j_*^17^. The SEPGG studies have revealed interesting results such as cyclic dominance^25^,^27^, transition nature^26^, and payoff distribution^28^. The effects of underlying topology on the SEPGG properties^17^,^28^,^29^,^30^,^31^,^32^,^33^ have also been found, such as the spatial reciprocity on diluted networks^34^ and multiplex networks^35^,^36^,^37^,^38^,^39^,^40^.

Since the SEPGG on regular lattices and sparse networks has considered only local interactions, the number of participants in a unit game centered at a node *i* cannot exceed *k_i_* + 1, where *k_i_* is the degree (or coordination number) of *i*. Thus, the SEPGG on sparse networks is hardly a theoretical model of real-life examples with very large participants such as taxes, provision levels, tolls, user fees, etc.^48^. Such cases involving public resources which anyone can overuse can be mapped into “tragedy of the commons” problem^49^,^50^. However, SEPGG in which all agents participate in a unit game has been rarely studied. Thus, we focus on SEPGG with very large participants.

In this report, the SEPGG with three strategies on a complete graph (CG) and dense complex networks is considered to understand the SEPGG with large participants. The CG is a simple undirected graph in which any node on the graph is linked to all other nodes. Thus, the number of links on the CG is *N*(*N* − 1)/2, where *N* is the number of nodes. In the SEPGG on the CG, all agents participate in a unit game. From analytically exact rate equations of the SEPGG on the CG, two stationary states depending on *r* and *N* are found. The state with only *L* agents (or *L*-state) is stable for low 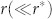. The state with only *D* agents (or *D*-state) is stable for high 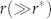. r* at which the crossover from the *L*-state to the *D*-state occurs is analytically obtained and also confirmed by numerical simulation. In the SEPGG on the CG, a *C*-dominant state cannot be stable even for very high *r*. These stationary states on the CG are very peculiar compared to the *C*-dominant state (or *C*-state) on regular lattices and sparse networks for very high *r*^28^,^30^,^31^,^32^,^33^. The *L*-state on the CG is also very peculiar in the sense that the *L*-state occurs only for *σ* > *c*(*r* − 1) in the PGG game with the well-mixed population^26^, whereas the *L*-state on the CG occurs even when 0 < *σ* < *c*(*r* − 1) or *r* is quite high.

More specifically, the time evolution of the SEPGG on the CG for high *r* is shown to have the following stages. In early time, the numbers of both *C* and *L* agents decrease, whereas the number of *D* agents hardly varies. Eventually, the *D*-state becomes stable. Hence, the time evolution of the SEPGG for high *r* describes key processes to the “tragedy of the commons” very well^49^,^50^, because the key processes are the following processes: First, the most of agents overuse the public resource in the commons as defector. Then, the overuse of the public resource will ruin it.

Ref. 26 revealed that the dominant state on sparse networks for high *r* is the *C*-state. Hence, we investigate crossover behaviors of the *L*-state or the *D*-state on dense networks such as the CG to a *C*-state on sparse networks by numerical simulation. For low *r*, first the crossover from the *L*-state to a *D*-state occurs, and the *D*-state successively crosses over to a *C*-state as mean-degree 〈*k*〉 decreases. Furthermore, the *D*-state for moderate 〈*k*〉 remains even in the limit *N* → ∞. We also quantitatively find that cooperation gradually increases as the number of participants or 〈*k*〉 decreases, which is the origin of two crossovers. Hence, the crossovers for low *r* describe how the enhanced cooperation on sparse networks with low 〈*k*〉 overcomes “tragedy of the commons”, resulting in the *C*-state. For high *r* the direct crossover from the D-state to the *C*-state occurs. This direct crossover is nearly the same as that from the *D*-state to the *C*-state for low *r*.

## Results

### SEPGG on the complete graph

From *f_ij_* in Eq. (11) using {*P_i_*} on the CG, exact rate equations of densities on the CG are written as
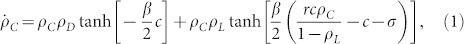

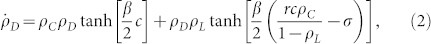
and
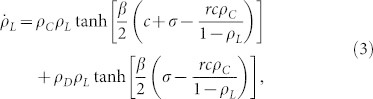
where 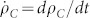, etc.

To obtain stationary states from general initial configurations with 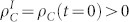, 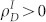 and 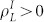, early time behaviors of *ρ_C_*, *ρ_D_*, and *ρ_L_* must be considered. Early time behaviors of *ρ_C_*, *ρ_D_*, and *ρ_L_* are determined based on competition between two terms of Eqs. (1)–(3), respectively. As *ρ_C_ρ_D_* tanh(−*βc*/2) ≤ 0 in Eq. (1) and *ρ_C_ρ_D_* tanh(*βc*/2) ≥ 0 in Eq. (2) for any non-negative *ρ_C_*, *ρ_D_*, *β* and *c*, two distinctive steady states are achievable depending on the value of *ρ_C_*. When 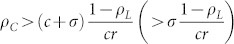, 
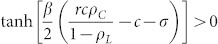
 in Eq. (1), 
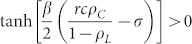
 in Eq. (2), and 
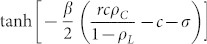
 in Eq. (3). Thus, 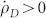 and 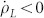, which make 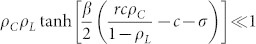 in Eq. (1) and 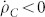 after some time. From these relations we find that the state of {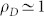, 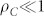, 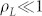} appears when 
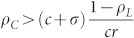
. Similarly, when 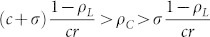, 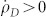 and 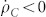, which also make 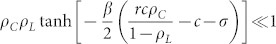 in Eq. (3) and 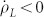 after some time. As a result, when 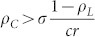, the state of {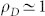, 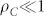, 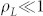} appears. We call this state the *D*-state. In contrast, when 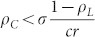, 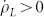 and 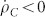, which make 
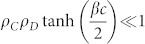
 in Eq. (2) and 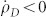 after some time. Thus, the state of {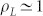, 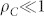, 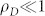} appears. We call this state the *L*-state. As the *D*-state or the *L*-state appears depending on the condition 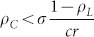, we now examine the stability of the *D*-state based on rate equations (1)–(3). If the *D*-state is unstable, the *L*-state should be stable.

In the D-state with {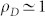, 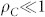, 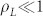}, the rate equation (1) becomes
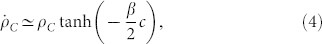
because 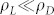. By solving Eq. (4) for time *t*, we obtain
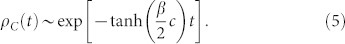
Similarly, the rate equation (3) also becomes
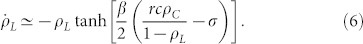
When 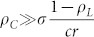, 
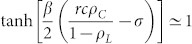
 and
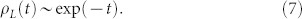
As *ρ_C_* decreases with *t*, the condition 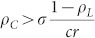 for the *D*-state breaks down for *t* > t*. From the Eq. (5) and the condition 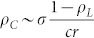 with 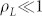, 
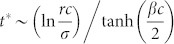
. Therefore, on the CG with *N* → ∞, the *L*-state is the only stationary state. However, on the CG with finite *N*, the nonzero-minimum of *ρ_L_* is 1/*N* and thus *ρ_L_* = 0 if *ρ_L_*(*t*) < 1/*N*. Therefore, if 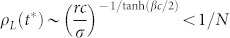, then *ρ_L_*(*t* > t*) = 0 and the *D*-state is still the stationary state. These results mean that the SEPGG on the CG with finite *N* has the following stationary state. For 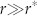, the *D*-state becomes stable, where
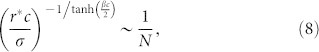
or
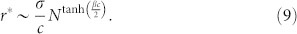
More specifically, this *D*-state for high *r* or 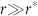 has never been found on regular lattices and sparse networks. As emphasized in our introductory remarks, this state also describes “tragedy of the commons” very well. In contrast, for 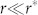, the *L*-state becomes stable. This *L*-state for 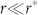 has never been found on regular lattices and sparse networks either. The *L*-state is also anomalous and surprising, because no body remains as an active participant in the PGG for 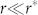. No *C*-dominant stationary state is found on the CG even for high *r*. Compared to the *C*-dominant stationary states on a square lattice^17^,^26^ and on sparse networks^28^,^30^,^31^,^32^,^33^ for high *r*, the stationary states on the CG are unique and anomalous.

**Figure 1:**
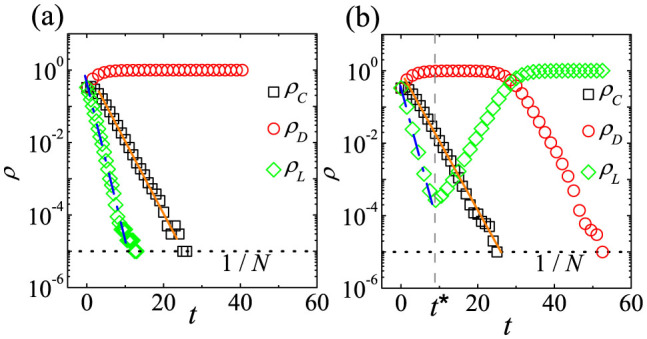
Simulation results of the SEPGG on the CG. Plots of *ρ_C_*(*t*), *ρ_D_*(*t*), and *ρ_L_*(*t*) of the SEPGG with *c* = 1, *σ* = 1, and *β* = 1 from a single simulation run with *N* = 10^5^. The dotted horizontal line denotes the value of 1/*N*. (a) When *r* = 2000, the stationary *D*-state appears. By fitting the data to Eqs. (5) and (7), *ρ_C_* ~ exp(~*α_C_t*) with 
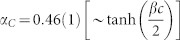
 (solid line) and *ρ_L_* ~ exp(−*α_L_t*) with *α_L_* = 1.00(2)(~1.0) (dash-dotted line) are obtained. (b) When *r* = 60, the stationary *L*-state eventually appears. The vertical dashed line denotes the value of 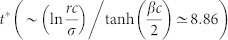. By the fitting, *ρ_C_* ~ exp(−*α_C_t*) with 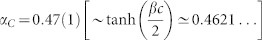 (solid line) and *ρ_L_* ~ exp(−*α_L_t*) with *α_L_* = 0.97(4)(~1.0) (dash-dotted line) are obtained for *t* < t*.

In Fig. 1, *ρ_C_*(*t*), *ρ_D_*(*t*), and *ρ_L_*(*t*) from a single run of simulation on the CG with *N* = 10^5^ are plotted. *ρ_C_*(*t*) and *ρ_L_*(*t*) decay exponentially in the early time regardless of the *r* value. For 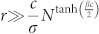, the time dependences of *ρ_C_*(*t*) and *ρ_L_*(*t*) are sustained throughout, and the stationary *D*-state eventually appears as shown in Fig. 1(a) (*r* = 2000). In contrast, when 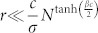, *ρ_L_*(*t*) increases after some time or for *t* > t* and the *L*-state eventually appears as shown in Fig. 1(b) (*r* = 60). Hence, simulation data presented in Fig. 1 exactly reproduce the analytical results of rate equations (1)–(3). More specifically, early time behaviors of *ρ_L_* ~ exp(−*t*) and 
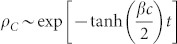
 are confirmed by fittings to simulation data as shown in Figs. 1(a) and 1(b). Furthermore, the crossover time t* for *r* = 60 is t* = 8.86 in Fig. 1(b), which is nearly identical to t* obtained from 
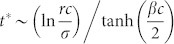
.

When 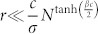 and in the limit of *N* → ∞, the time dependences of *ρ_C_*, *ρ_D_* and *ρ_L_* on the CG shown in Fig. 1(b) effectively present the process to the anomalous *L*-state with no active participants. The process means the following three steps. First, most agents defect one another. *C* then changes his strategy to *D*, and *ρ_C_*(*t*) decreases. Thus, *D* cannot receive enough payoff^50^, causing *ρ_D_*(*t*) to decrease and *ρ_L_*(*t*) to increase. Finally, most agents become *L*, as no one remains in the commons. Consequently, the stationary *L*-state eventually appears for 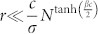.

**Figure 2:**
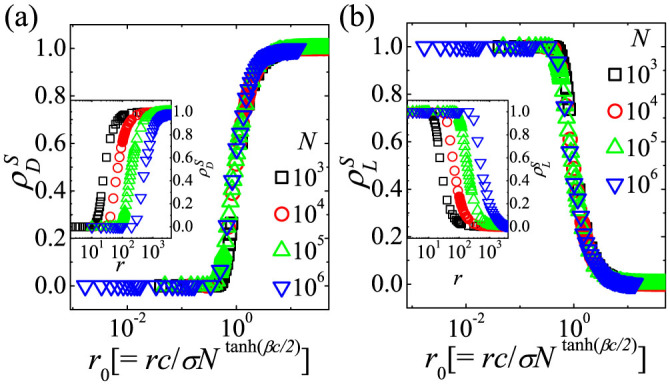
Simulation results of the SEPGG on the CG for various *r* and *N*. Plots of (a) 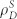 and (b) 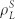 against 
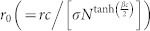
 for *N* = 10^3^, 10^4^, 10^5^, and 10^6^. *c* = 1, *σ* = 1, and *β* = 1 are used. Inset of (a): Plots of 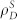 against *r*. Inset of (b): Plots of 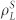 against *r*.

To analyze the dependence of stationary states on the multiplication factor *r*, 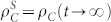, 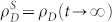, and 
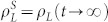
 are obtained from simulations for various *N* and *r* by averaging over 1,000 realizations. Simulation results of 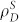 and 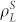 for various *N* and *r* are shown in the insets of Fig. 2. As shown in insets of Fig. 2, the crossover value of *r*, i.e., r*, from the stationary *L*-state to the stationary *D*-state increases with *N* as expected from Eq. (9). More specifically, 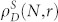 and 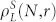 in Fig. 2 exactly depend on the single scaling parameter *r*_0_ defined as 
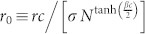
. The scaling behaviors confirm that the *L*-state crosses over to the *D*-state at 
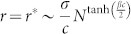
 as Eq. (9).

### Crossover from the behavior on dense networks to that on sparse networks

A dense network is a network in which the mean-degree 〈*k*〉 satisfies 〈*k*〉 ∝ *N*^51^. For example, the CG is a typical dense network, as 〈*k*〉 = *N* − 1 in the CG. In a sparse network, 〈*k*〉 = *finite*^51^. In the SEPGG on the CG, either the *L*-state or the *D*-state is stable depending on *r* and *N* and the *C*-dominant state cannot be stable. In contrast, the *C*-dominant state is stable for relatively high *r* in the SEPGG on sparse networks such as random networks^30^,^33^ and two dimensional square lattices^17^,^26^. Therefore, it is interesting to study how crossover from the *L*-state and the *D*-state on dense networks to the *C*-dominant state on sparse networks occurs for given values of *r* and *N*.

**Figure 3:**
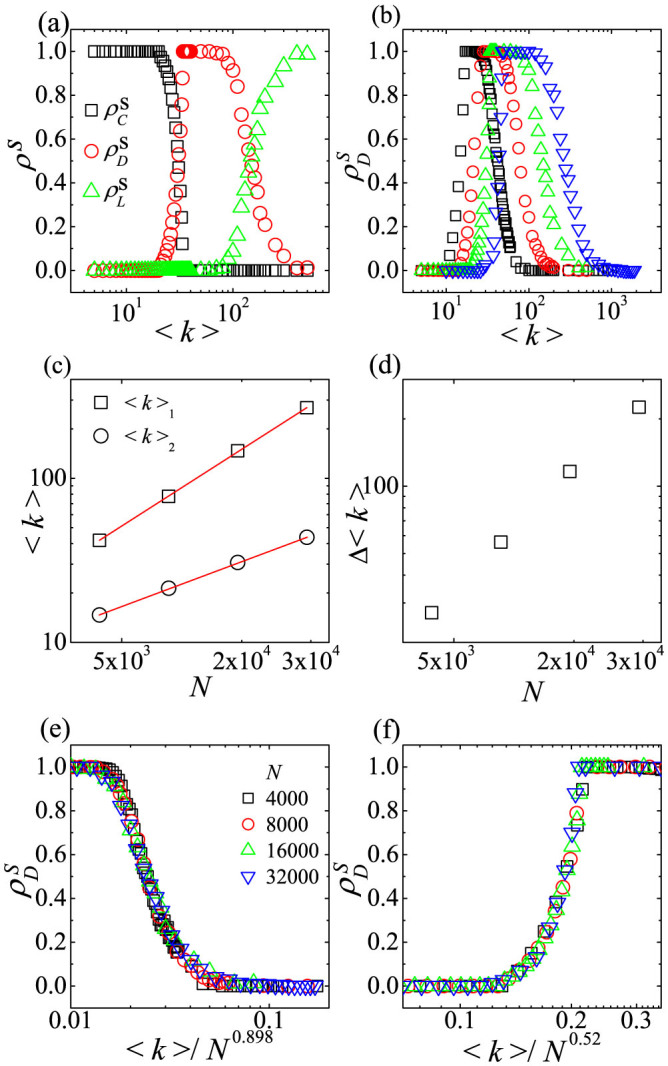
Simulation results of the SEPGG on random networks for 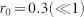. (a) Plots of 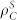, 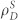, and 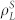 against 〈*k*〉 for *N* = 16000. *c* = 1, *σ* = 1, and *β* = 1 are used. (b) Plots of 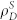 against 〈*k*〉 for *N* = 4000, 8000, 16000, and 32000. Here, 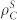 and 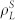 are not shown, because 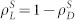 for high 〈*k*〉 and 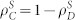 for low 〈*k*〉. (c) Plots of 〈*k*〉_1_ and 〈*k*〉_2_ against *N*. The straight lines denotes fittings of 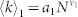 with 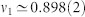 and 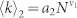 with 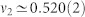 to corresponding data. (d) Plot of Δ 〈*k*〉 (≡〈*k*〉_1_ − 〈*k*〉_2_) against *N*. (e) Plot of 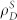 against 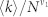 with *ν*_1_ in (c). (f) Plot of 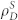 against 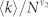 with *ν*_2_ in (c).

We first investigate how the *L*-state on dense networks crosses over to the *C*-dominant state on sparse networks. Since the *L*-state is stable for low *r*_0_ on the CG as shown in Fig. 2, the crossover behaviors for low *r*_0_ are studied by simulations on random networks with 〈*k*〉. For a given *N* and 〈*k*〉, 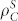, 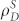, and 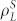 are obtained by averaging over 2,000 realizations. Typical crossover behaviors for *r*_0_ = 0.3 are shown in Fig. 3. As shown in Fig. 3(a), two crossovers occur successively as 〈*k*〉 decreases. The *L*-state is stable when 〈*k*〉 is quite high. The *C*-state of {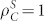, 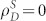, 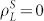} is stable when 〈*k*〉 is low enough. For moderate 〈*k*〉 the *D*-state is stable. Therefore, for low *r*_0_, the stationary state is first changed from the *L*-state to a *D*-state and crossover from the *D*-state to a *C*-state occurs as 〈*k*〉 decreases.

**Figure 4:**
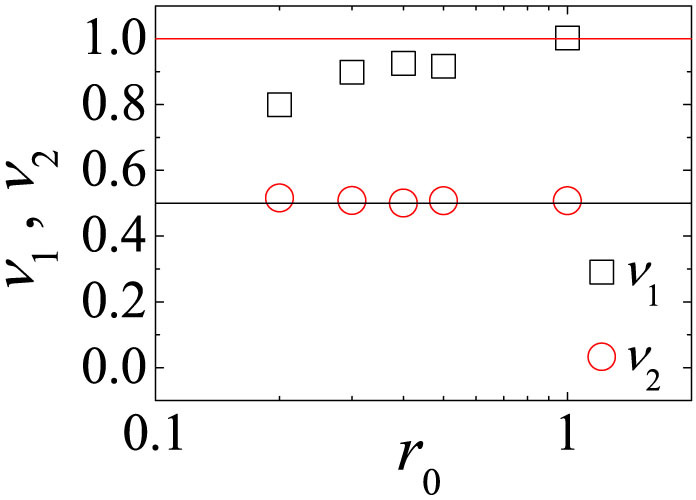
Plots of exponents *ν*_1_ and *ν*_2_ against *r*_0_. *c* = 1, *σ* = 1, and *β* = 1 are used. In the limit *N* → ∞, the *D*-state for moderate 〈*k*〉 is stable, because *ν*_1_ > *ν*_2_.

The stability of the *D*-state for moderate 〈*k*〉 in the limit *N* → ∞ is studied using the following methods. From simulation data of 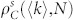, 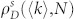 and 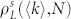 as in Figs. 3(a) and 3(b), we first obtain 〈*k*〉_1_ at which relations 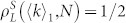 and 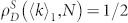 hold simultaneously. We also obtain 〈*k*〉_2_ at which 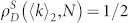 and 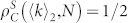 hold. For example, dependences of 〈*k*〉_1_ and 〈*k*〉_2_ on *N* for *r*_0_ = 0.3 are shown in Fig. 3(c). The dependence of Δ 〈*k*〉 (≡〈*k*〉_1_ − 〈*k*〉_2_) is also shown in Fig. 3(d). As shown in Fig. 3(d), Δ 〈*k*〉 increases monotonically with *N*, guaranteeing the stability of the *D*-state for moderate 〈*k*〉 in the limit *N* → ∞. Furthermore, as shown in Fig. 3(c), 〈*k*〉_1_ and 〈*k*〉_2_ satisfy power laws 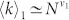 and 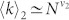. By fitting these power laws to data presented in Fig. 3(c), crossover exponents are obtained as *ν*_1_ = 0.898(2), *ν*_2_ = 0.520(2). The result *ν*_1_ > *ν*_2_ also guarantees the stability of the *D*-state for moderate 〈*k*〉. The crossover property from the *L*-state to the *D*-state presented in Fig. 3(b) is adequately described by the single exponent *ν*_1_ obtained in Fig. 3(c). 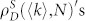 for higher 〈*k*〉 and various *N* are plotted against the scaling variable 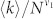 with the obtained *ν*_1_ as in Fig. 3(e), which shows that 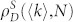 for higher 〈*k*〉 is a function of the single scaling variable 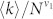. As shown in Fig. 3(f), crossover from the *D*-state to the *C*-state also satisfies the scaling property that 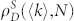 for lower 〈*k*〉 is a function of the single scaling variable 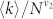 with the obtained exponent *ν*_2_. Using the same method *ν*_1_'s and *ν*_2_'s for various low *r*_0_(<1) are obtained as shown in Fig. 4. Because *ν*_1_ > *ν*_2_ in Fig. 4, the *D*-state for moderate 〈*k*〉 and low *r*_0_(<1) is stable in the limit *N* → ∞.

Furthermore, the dependences of 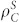, 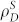, and 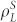 on 〈*k*〉 for low *r*_0_ in Fig. 3(a) are quite similar to the time dependences of *ρ_C_*(*t*), *ρ_D_*(*t*), and *ρ_L_*(*t*) on the CG for low *r*_0_ shown in Fig. 1(b). In Fig. 1(b), initially there are enough *C*s. As *t* increases, *D* governs the system. Finally *L* dominates, because *D* cannot receive enough payoff. Likewise, in Fig. 3(a), for low 〈*k*〉 there are also enough *C*s. For moderate 〈*k*〉 *D* governs the system. When 〈*k*〉 becomes high enough, *L* dominates. Hence, it is very interesting to compare dynamical behaviors on the CG to static crossover behaviors depending on 〈*k*〉.

**Figure 5:**
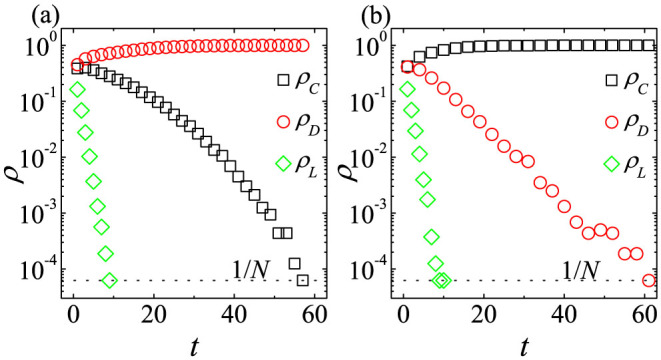
Time dependence of *ρ_C_*(*t*), *ρ_D_*(*t*), and *ρ_L_*(*t*) on random networks with *N* = 16000 for *r*_0_ = 0.3. Plots of *ρ_C_*(*t*), *ρ_D_*(*t*), and *ρ_L_*(*t*) (a) for moderate 〈*k*〉 ( = 30) and (b) for low 〈*k*〉 ( = 10). (a) For moderate 〈*k*〉 ( = 30), *ρ_D_* increases with *t*, whereas *ρ_C_* and *ρ_L_* decreases. Finally, the stationary *D*-state emerges. (b) For low 〈*k*〉 ( = 10), *ρ_C_* increases with *t*, whereas *ρ_D_* and *ρ_L_* decreases. Finally, the stationary *C*-state appears. The time dependences for high 〈*k*〉 are not shown, because they are nearly the same as those shown in Fig. 1(b).

We thus now focus on the time dependence of *ρ_C_*(*t*), *ρ_D_*(*t*), and *ρ_L_*(*t*) for various 〈*k*〉 to understand crossover behaviors for low *r*_0_ in Fig. 3(a). The time dependences of *ρ_C_*, *ρ_D_*, and *ρ_L_* for moderate 〈*k*〉 are shown in Fig. 5(a), and those for low 〈*k*〉 are shown in Fig. 5(b). For high 〈*k*〉, the time dependence is nearly identical to that on the CG shown in Fig. 1(b). For moderate 〈*k*〉 and high 〈*k*〉, *ρ_C_* and *ρ_L_* decrease, but *ρ_D_* increases in early time. However, the stationary state is strongly affected by the subsequent time dependence of *ρ_C_*. If 〈*k*〉 is quite high or if 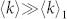, *ρ_C_* decays quickly and *ρ_D_* cannot receive enough payoff. As a result, *ρ_L_* increases for *t* > t* and the stationary *L*-state appears as explained in Fig. 1(b). In contrast, for moderate 〈*k*〉 or 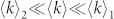, *ρ_C_*(*t*) decreases relatively slowly, and *ρ_L_*(*t*) never have a chance to increase reversely before the time at which *ρ_L_*(*t*) ≤ 1/*N* [see Fig. 5(a)]. This means that the cooperation is effectively enhanced for moderate 〈*k*〉 and *D* receives enough payoff until *L* disappears due to the enhanced cooperation. This first crossover is quite similar to the crossover from the *L*-state in Fig. 1(b) to *D*-state in Fig. 1(a) on the CG. For low 〈*k*〉 or 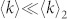, *ρ_C_*(*t*) never decreases as on sparse networks^28^,^30^,^31^,^32^,^33^ [see Figs. 5(b)], and 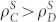. Hence, the crossover from the *D*-state to the *C*-state (or *C*-dominant state) occurs for 〈*k*〉 ~ 〈*k*〉_2_ as 〈*k*〉 decreases.

The two crossovers for low *r*_0_ thus derive from a gradual increase of cooperation as the number of participants (or 〈*k*〉) decreases. Therefore, the crossovers that describe the disappearance of both the anomalous state with no active participants and “tragedy of the commons” quantitatively show that agents in the larger group hardly cooperate relative to those in the smaller group^45^,^46^. However, this dependence on the group size is not necessarily accurate, because a recent study on PGG^44^ reported that increasing the group size does not necessarily lead to mean-field behaviors.

**Figure 6:**
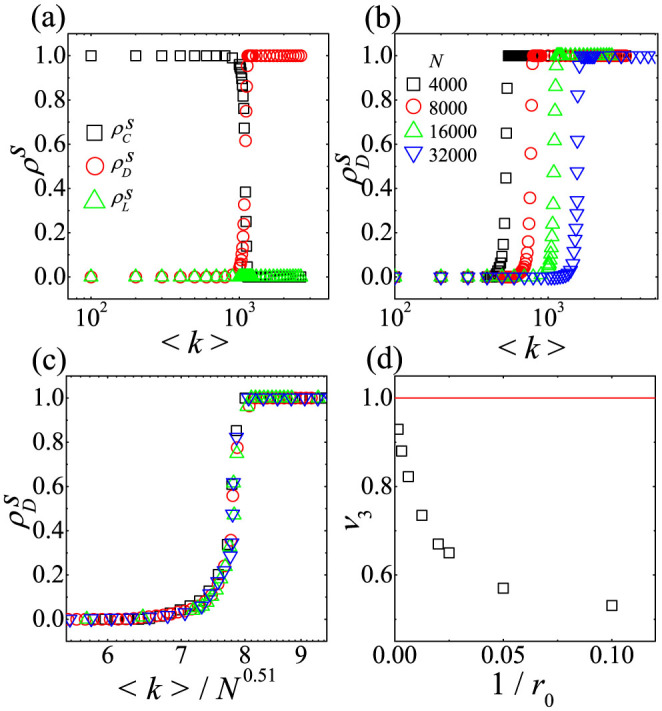
Simulation results of the SEPGG on random networks for 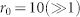. (a) Plots of 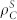, 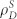, and 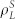 against 〈*k*〉 for *N* = 16000. *c* = 1, *σ* = 1, and *β* = 1 are used. The stationary state is changed from the *D*-state to a *C*-state as 〈*k*〉 decreases. 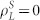 for any 〈*k*〉. (b) Plots of 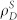 against 〈*k*〉 for *N* = 4000, 8000, 16000, and 32000. (c) Plot of 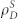 against 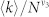 with 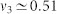. (d) Plot of *ν*_3_ against 1/*r*_0_.

Finally, we study the crossover from the *D*-state to a *C*-state for high *r*_0_(>1). Typical crossover behaviors for high *r*_0_ are shown in Fig. 6(a). As shown in Fig. 6(a), for high *r*_0_( = 10), the *D*-state is stable when 〈*k*〉 is quite high. The *C*-state is stable when 〈*k*〉 is low enough. Therefore, for high *r*_0_, the direct crossover from the *D*-state to the *C*-state occurs as 〈*k*〉 decreases. To analyze the dependence of this direct crossover on *N*, 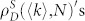 for various *N* are obtained by simulation as shown in Fig. 6(b). The dependence of the direct crossover on *N* can be obtained by the ansatz 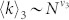, where at 〈*k*〉_3_ both 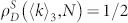 and 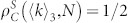 hold. From the dependence of 〈*k*〉_3_ on *N*, 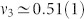 is obtained for *r*_0_ = 10. This direct crossover satisfies the scaling property that 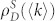 is a function of the single scaling variable 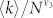 with *ν*_3_ = 0.51. As shown in Fig. 6(d), *ν*_3_'s for various high *r*_0_(>1) are obtained using the same method. The data in Fig. 6(d) show that the value of *ν*_3_ increases as *r*_0_ increases. As the *D*-state is always stable on the CG or dense networks with 〈*k*〉 ∝ *N*, the upper bound of *ν*_3_ should be equal to 1. We also confirm that the time dependences of *ρ_C_*(*t*), *ρ_D_*(*t*), and *ρ_L_*(*t*) for high *r*_0_ are nearly the same as those in Fig. 1(a) for high 〈*k*〉 and as those in Fig. 5(b) for low 〈*k*〉, respectively. Hence, this direct crossover is nearly identical to the second crossover from the *D*-state to the *C*-state for low *r*_0_.

## Discussion

In summary, we have studied the SEPGG on the CG and complex dense networks to understand behaviors of the SEPGG with very large participants. By analyses of the rate equations, we have shown that the *L*-state of {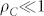, 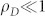, 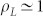} is stable on the CG for *r* < r* with 
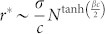
. In contrast, the *D*-state of {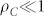, 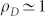, 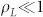}, representing “tragedy of the commons”, is stable for *r* > r*. These analytic results on the CG have been confirmed by simulation.

We have also studied crossover behaviors from the *L*-state or the *D*-state on dense networks to the *C*-dominate state on sparse networks by numerical simulation on random networks with a mean degree 〈*k*〉. For *r* < r*, the *L*-state first crosses over to a *D*-state, and successively this *D*-state crosses over to a *C*-state as 〈*k*〉 decreases. We have investigated the dependence of the crossovers on *N* for low *r*_0_ using the ansatz 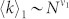 and 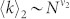, where the *L*-state is stable for 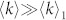, the *D*-state is stable for 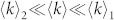, and the *C*-state is stable for 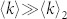. From the numerical simulations, *ν*_1_ and *ν*_2_ have been obtained. Since *ν*_1_ > *ν*_2_ for *r* < r*, we have found that the *D*-state for moderate 〈*k*〉 is stable even in the limit *N* → ∞. We have also studied the time dependences of *ρ_C_*, *ρ_D_*, and *ρ_L_* on random networks with 〈*k*〉 to understand the crossover behaviors for *r* < r*. For moderate 〈*k*〉, the *D*-state is stable, because *ρ_C_* decreases relatively slowly. For low 〈*k*〉, cooperation is enhanced and the *C*-state is stable. The two crossovers for *r* < r* derive from a gradual increase of cooperation as the number of participants (or 〈*k*〉) decreases. The crossovers thus show how the enhanced cooperation on sparse networks with low 〈*k*〉 produces the *C*-state, overcoming both the anomalous state with no active participants and “tragedy of the commons” for low *r*_0_.

For high *r*_0_, the *D*-state is stable when 〈*k*〉 is high. The *C*-state is stable when 〈*k*〉 is low. Therefore, for high *r*_0_, the direct crossover from the *D*-state to the *C*-state occurs as 〈*k*〉 decreases. The dependence of the direct crossover on *N* has been also analyzed by the ansatz 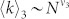, where the *D*-state appears for 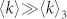 and the *C*-state appears for 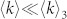. From the numerical simulations, *ν*_3_ has been obtained. The value of *ν*_3_ increases to 1 as *r*_0_ increases, because the *D*-state always appears on the CG or dense networks with 〈*k*〉 ∝ *N*. The crossovers thus describe how the enhanced cooperation on sparse networks with low 〈*k*〉 overcomes “tragedy of the commons” and makes the *C*-state for high *r*_0_.

Finally, the cyclic dominance in Ref. 25 can also be found for very low *r* and 〈*k*〉. For example, for *r*_0_ = 0.1, the crossover from the *C*-state to the cyclic dominance occurs at 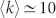 on the network with the size *N* = 10^4^. This crossover behavior is not explained quantitatively here, because the crossover occurs only on sparse networks.

## Methods

Let us define the SEPGG model on a given graph or network in detail. Each agent is assigned to a node on the network. Variable *s_i_* of the agent on node *i* represents the strategy of *i*. The *s_i_* is a cooperator (*C*), defector (*D*) or loner (*L*). The number of agents with a given strategy is denoted as 
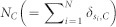
, 
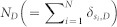
, and 
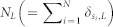
, where *N* is the size of the network.

In each update of SEPGG on the network, an agent *i* is randomly selected. Then, the payoff *P_i_* of *i* depends on the strategies of *k_i_* + 1 participants, where *k_i_* is the degree of *i*. If *n_i_*_,*C*_ is the number of agents with *C*, *n_i_*_,*D*_ is the number of agents with *D*, and *n_i_*_,*L*_ is the number of agents with *L* among the *k_i_* + 1 participants, *n_i_*_,*C*_ + *n_i_*_,*D*_ + *n_i_*_,*L*_ = *k_i_* + 1. *P_i_* is thus given by
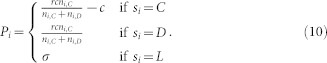
Here, *c* is the cost contributed by a *C* to the common pool, *r*(>1) is the multiplication factor and *σ* is the fixed payoff of a *L*^26^. We impose the condition 0 < *σ* < *c*(*r* − 1) as in Ref. 25. Even if only one active participant remains, the payoff of the agent still follows Eq. (10). Then, the strategy of *i* is updated through the comparison of *P_i_* with *P_j_* of a randomly selected neighbor *j* among *k_i_* neighbors in order to select a better strategy. If *s_i_* ≠ *s_j_*, the agent *i* stochastically adopts the strategy *s_j_* of the neighbor *j* with transition probability *f_ij_*. We use
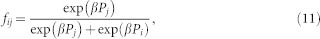
as in Ref. 17. Here *β*(≥0) controls the amount of noise. In each update of SEPGG, the payoffs in *f_ij_* of Eq. (11) on regular lattices and sparse networks depend on the configuration of all the agents at the time of the update. In contrast, *P_i_* in *f_ij_* on the CG depends only on *s_i_* and *N_C_*, *N_D_*, and *N_L_*, of the strategies on the entire graph, because all agents participate in each unit game. The payoff {*P_i_*} on the CG is thus written as
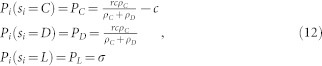
where the densities *ρ_C_*(≡*N_C_*/*N*), *ρ_D_*(≡*N_D_*/*N*), and *ρ_L_*(≡*N_L_*/*N*) are used. To confirm the analytic results, simulations are performed for various *N* and *r*. Here, we mainly report the results of simulations with 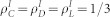, *c* = 1, *σ* = 1 and *β* = 1. Simulations with various combinations of 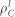, 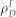, 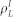, *c*, *σ* and *β* are tested and nearly identical results are obtained.

## Author Contributions

J.K., H.C., S.-H.Y. and Y.K. designed the study; H.C. performed the analytic calculation; J.K. performed the simulations and analyzed data; H.C., S.-H.Y. and Y.K. wrote the manuscript. All authors revised the manuscript.
